# Lack of Restoration *in Vivo* by K^+^-Channel Modulators of Jejunal Fluid Absorption after Heat Stable *Escherichia coli* Enterotoxin (STa) Challenge

**DOI:** 10.1155/2011/853686

**Published:** 2011-06-12

**Authors:** M. L. Lucas, L. C. Gilligan, C. C. Whitelaw, P. J. Wynne, J. D. Morrison

**Affiliations:** Laboratory of Gastrointestinal Physiology, School of Life Sciences, College of Medical, Veterinary and Life Sciences, Glasgow University, Glasgow G12 8QQ, Scotland, UK

## Abstract

Enhanced potassium ion permeability at the enterocyte basolateral membrane is assumed to facilitate sustained chloride ion and fluid secretion into the intestinal lumen during episodes of secretory diarrhoeal disease. To examine this concept *in vivo*, two potassium ion channel blockers and a channel opener were coperfused with *E. coli* heat stable STa enterotoxin to determine whether such compounds improved or worsened the inhibited fluid absorption. In the STa (80 ng/mL) challenged jejunal loop, the fluid absorption rate of 28.6 ± 5.8 (14) *μ*L/cm/hr was significantly below (*P* < .001) the normal rate of 98.8 ± 6.2 (17) *μ*L/cm/hr. Intraluminal (300 uM) glibenclamide added to STa perfused loops failed to improve the inhibited fluid absorption rate, which was 7.4 ± 3.2 (6) *μ*L/cm/hr on coperfusion with STa. Similarly, on coperfusion with 30 uM clotrimazole, the fluid absorption rate with STa present remained inhibited at 11.4 ± 7.0 (4) *μ*L/cm/hr. On coperfusion with intraluminal 1 uM cromakalim, STa reduced fluid absorption significantly (*P* < .02) to 24.7 ± 8.0 (10) *μ*L/cm/hr, no different from STa challenge in the absence of cromakalim. Infusion i.v. with these agents also failed to restore fluid absorption after STa challenge. These observations do not support the proposed potassium ion permeability event as a necessary corollary of enterotoxin-mediated secretion. This makes it unlikely that modulators of such permeability prevent enterocyte secretion in diarrhoeal disease.

## 1. Introduction

Fluid and electrolyte absorption is an essential function of the enterocytes of the small intestine. In addition, enterocytes are assumed also to be capable of fluid secretion into the intestine resulting from the transport of chloride ion into the lumen although this enterocyte origin of secretion has been questioned [1, 2]. Net fluid uptake is, therefore, regarded as fluid absorption opposed in part by an enterocyte secretory process that can be enhanced by bacterial enterotoxins to cause diarrhoeal disease. Chloride entry from the interstitial fluid into the enterocyte is achieved principally by the NKCC cotransporter with subsequent transit through the luminally sited CFTR channel. Given the adverse lumen to the enterocyte interior chemical concentration gradient, luminal exit of chloride ion can only arise through a favourable electrical gradient across the luminal face of the enterocyte. Maintenance of this membrane potential through modulation of basolateral potassium ion conductance is, therefore, assumed to be an important aspect of the secretory process.

Many *in vitro* studies of electrical events in cell systems after STa and cholera toxin challenge apparently confirm this aspect of the secretion model [3–5]. In contrast, there have been only two previous *in vivo* studies on potassium channel openers and blockers in the enterotoxin challenged small intestine, and the most detailed account seems to contradict the above model of the role of potassium ion channel opening. Reduced fluid absorption *in vivo* from the rat jejunum after STa challenge was partially restored by glibenclamide and also, less explicably, by cromakalim, the potassium channel opener [6]. The other *in vivo* study, a brief report on the pig intestine, indicated that the potassium channel blocker clotrimazole did not overcome the effect of STa challenge [7].

We report here on similar findings in the rat jejunum *in vivo* on the effect of clotrimazole, cromakalim, and glibenclamide on jejunal fluid absorption* in vivo *in the rat compromised by perfusion with *E. coli* STa enterotoxin. These experiments intended to determine whether or not K^+^-channel modulators administered *in vivo* could restore or even further reduce the effects of STa on fluid absorption, as has been hypothesised should occur on the basis of *in vitro* findings. Jejunum was chosen rather than ileum, as the jejunum has higher rates of fluid absorption that are susceptible to STa derangement. Ileum can be used, but absorption rates are about one half those in the jejunum. When tested *in vivo*, these potassium channel modulators had no effect on STa-inhibited fluid absorption, making it unlikely that they would be effective antidiarrhoeal compounds. In addition, the failure of potassium channel modulators to alter STa action adds to the available *in vivo* evidence against the enterocyte secretory model of diarrhoeal disease.

## 2. Methods

### 2.1. *In Vivo* Perfusion Procedures

All experiments complied with current UK legislation and were approved after internal ethical review. Fluid absorption from perfused *in vivo* rat jejunal loops was measured by a recirculation procedure [8] described in detail elsewhere [9]. Adult Sprague-Dawley female rats, anaesthetised with sodium pentobarbitone (70 mg/kg body weight *i.p*.) were tracheotomised to allow unimpeded breathing of room air. Anaesthesia was sustained by periodic *i.p*. dosage sufficient to maintain abolition of the hind limb flexor withdrawal reflex. Body temperature was kept at 37°C by a heating table controlled by rectal thermistor. Twenty five cm jejunal loops, starting ten cms below the ligament of Treitz, were perfused via PVC tubing at 1.8 mL/min by peristaltic pump (Crouzet 82344, UK) from a reservoir containing an initial volume of 25 mLs of perfusate maintained at 37°C. After three hours, the experiment was ended by pumping air into the loop and collecting all remaining perfusate in the reservoir. In addition, the loop was excised, and any fluid remaining in the loop was expelled by air with a 20-mL syringe into the reservoir under gravity drainage. Final recovered volume in the reservoir was measured to the nearest 0.1 mL. Loop length was measured by ruler after gently laying out the excised loop on the bench without stretching it. Fluid absorption as measured by this fluid recovery [10] method is the difference between the initial added and final recovered volumes after emptying the perfusate reservoir. It is presented here as microlitres of fluid per cm length of intestine per hour with positive values meaning absorption. Excised loops were weighed and also dried to constant weight in an oven at 100°C, enabling fluid uptake also to be standardised per milligram wet weight and per gram dry weight. After experiment, the animal was euthanised. The perfusate was 100 mM sodium bicarbonate solution and 54 mM sodium chloride solution to maintain isotonicity, with 80 ng/mL STa as required. Absorption per gram wet and per dry weight was calculated but are not presented, as change in standardisation did not alter any outcome.

### 2.2. Mean Arterial Blood Pressure Measurements

In preliminary experiments to confirm the activity of the potassium ion channel agents, arterial blood pressure was measured via a carotid cannula (Portex, “pink” o.d. 0.9 mm) using a Statham 23 B pressure transducer coupled via a 1401 CED ac/dc board to a Dell Pentium and recorded by the “Chart” data capture programme (freeware from J Dempster, University of Strathclyde, Scotland, UK). Where these drugs were given as part of the perfusion protocol for assessing their effect on absorption, blood pressure was measured with a Harvard pressure transducer, coupled to a Toshiba lap-top computer and recorded using Spike 2 for data capture via the CED 1401 interface (Cambridge Instruments, Cambs, UK). Heart rate and respiration frequency (from the Traube-Hering waves) were also measured from the blood pressure record.

### 2.3. Administration of Potassium Channel Active Agents

Cromakalim and clotrimazole were dispensed from stock solutions of 5 mM in ethanol. Experiments were done using ethanol vehicle to confirm that this was without effect on the reduced fluid absorption caused by STa. Glibenclamide was dispensed from a 300 mM stock solution in DMSO (dimethyl sulphoxide). Similar control experiments with DMSO confirmed that vehicle alone had no effect on STa action.

Cromakalim and glibenclamide had been previously identified as being antisecretory [6], using a procedure with the relatively short perfusion time of forty-five minutes. These substances were investigated in recirculated loops for a longer time to examine any sustained ability to inhibit STa action. Because of the longer experimental duration, these drugs could not be administered exactly as in the Schirgi-Degen protocol, since cromakalim was infused intravenously at a rate of 63.5 ug/kg/min for 45 minutes only, whereas longer infusion at that rate eventually causes fatal hypotension. Cromakalim and clotrimazole were, therefore, administered for the first 45 minutes as per the Schirgi-Degen protocol. This continued until mean arterial blood pressure fell by 30% below the initial preinfusion value, at which point dosing stopped and was resumed only when mean arterial blood pressure again exceeded this value. Consistency was, therefore, achieved by maintaining a relatively constant extent of depressor activity. The accumulated dosage rates of 2.85 ± 0.30 (5) mg/kg/hr for clotrimazole and 2.72 ± 0.32 (5) mg/kg/hr for cromakalim were in fact very close to the Schirgi-Degen protocol rate for cromakalim. As glibenclamide has a moderate pressor effect [11], it was given by periodic i.v. injection at a rate of  7.0 mg/kg/hr, close to the 9.6 mg/kg/hr rate given intra-arterially in the Schirgi-Degen protocol.

### 2.4. *In Vitro* Ileum Motility Assay

The ability of the potassium channel active agents to alter intestinal smooth muscle function was also tested in separate *in vitro* experiments by measuring changes in longitudinal tension in the rabbit ileum mounted in a Burn-Dale apparatus and maintained in aerated Krebs-Ringer at 37°C. Tension was measured by a Harvard instruments tension transducer via an ac/dc converter board recorded by a Dell Pentium PC using the “Chart” data capture programme. Rabbit ileal tissue became available during the time of this study but was not purposefully selected as a test assay for motility changes.

### 2.5. Source of Chemicals

*E. coli STa* and other chemicals were purchased from Sigma Chemical Co (Poole, Dorset, UK). For some of the later experiments, STa from a P16 strain was used at an equivalent dose after the synthetic peptide became unavailable. There were no significant differences in the effect of STa on fluid absorption, since the reduced fluid absorption of 23.5 ± 8.5 (8) *μ*L/cm/hr after P16 STa exposure did not differ significantly from the rate of 33.2 ± 8.6 (9) *μ*L/cm/hr after Sigma STa exposure. Sagatal (sodium pentobarbitone) was obtained from Rhone Merieux Ltd (Harrow, London, UK).

### 2.6. Statistical Analysis of Data

Results are given as the mean and standard error of the mean, followed by the number of experiments, equal to the number of animals, in parenthesis. Implementation of all statistical analysis was done using BMDP [12]. Where required, comparison of means was by analysis of variance within BMDP P1V that calculated Student's *t* values after Dunnett's [13] correction for multiple comparisons.

## 3. Results

### 3.1. Preliminary Experiments

The potassium channel active agents used to attempt to prevent the effect of STa on fluid absorption were tested for their known pressor effects on mean arterial blood pressure *in vivo* and on smooth muscle tension *in vitro*. This was intended to confirm, by using the same stock solutions, that the drugs introduced into the small intestine had demonstrable effects on other organ systems. In a representative group of untreated animals, systolic/diastolic pressure was 128.3 ± 11.7 (6) over 112.0 ± 13.8 (6) mm Hg, giving a mean arterial blood pressure of 117.4 ± 13.6 (6) mm Hg. Intravenous glibenclamide (Table 1) as a bolus dose of 1.5 mg/kg significantly (*P* < .01) increased systolic by 21.7 ± 5.8 (7), diastolic by 20.3 ± 4.7 (7) and mean arterial blood pressure by 20.8 ± 4.8 (7) mm Hg. Clotrimazole as a bolus dose of 1 mg/kg depressed mean systolic pressure significantly (*P* < .01) to 87.2 ± 7.6 (6) mm Hg, the diastolic pressure to 66.3 ± 5.6 (6) mm Hg and mean arterial blood pressure to 73.3 ± 13.1 (6) mm Hg. Clotrimazole administration caused marked bradycardia in some animals for 20 seconds or longer, coupled with a compensatory increase in pulse pressure, as predicted by the Frank-Starling law of the heart. Cromakalim at a dose of 24 ug/kg depressed mean arterial blood pressure to 58 ± 5 (3) which was significantly less (*P* < .02) by 38 ± 5 (3) mm Hg pressure than control values. A higher dose of 60 ug/kg depressed mean arterial blood pressure even further to 42 ± 10 (3) mm Hg. In contrast to clotrimazole, there were no episodes of bradycardia with cromakalim or late pressor effects as with glibenclamide. However, it was evident that all three drugs prepared from the same stock solutions used to perfuse the small intestine showed demonstrable effects on the cardiovascular system.

The ability of these compounds to affect smooth muscle tension was also determined *in vitro* in experiments on rabbit ileum incubated in Krebs-Ringer solution. Confirming the effects on vascular smooth muscle tone *in vivo*, 7 uM cromakalim and 100 uM clotrimazole reduced intestinal smooth muscle longitudinal tension (Table 2). Basal tension in four preparations was 2.5 ± 0.9 (4) grams rising to a peak of 5.9 ± 0.8 (4) grams, giving an average tension of 3.7 ± 0.5 (4) grams, a tension amplitude of 3.4 ± 0.9 grams, with a mean rate of contraction of 13.9 ± 1.1 contractions per minute. Clotrimazole had no effect on the basal tension but reduced peak tension significantly (*P* < .001) to 4.6 ± 0.6 (4) grams and reduced average tension and tension amplitude, without affecting the rate of contraction. Cromokalim similarly reduced (*P* < .05) tension amplitude from 5.1 ± 1.3 (3) to 2.8 ± 1.3 (3) grams without altering frequency of contraction. In contrast, 50 uM glibenclamide significantly (*P* < .001) increased the basal tension from 2.0 ± 0.5 (3) to 3.1 ± 0.6 (3) grams and also had no effect on frequency of contraction. These results are not novel and served only to confirm the blood pressure results in that clotrimazole and cromakalim reduced smooth muscle tone and glibenclamide showed a pressor action, as is well known and as others have found [11, 14, 15].

In a group of animals perfused luminally with STa, mean arterial blood pressure was 108 ± 11.7 (3) mm Hg at the onset of perfusion. After three hours perfusion, the mean arterial blood pressure in the STa perfused animals rose to 125 ± 5 (3) mm Hg, indicating that pressure was maintained and unaffected by intraluminal STa perfusion. In a similarly representative group perfused with STa, heart rate was 375 ± 10 (5) beats per minute, and the respiratory rate was 53 ± 5 (5) breaths per minute, with neither variable significantly changing over the perfusion period. Hence, three or more hours of perfusion under anaesthesia did not lead to respiratory or cardiovascular depression even when the lumen was perfused with* E. coli* STa.

### 3.2. Effect of Luminal and I.v. Glibenclamide on Luminal Fluid Absorption from the Proximal Jejunum after *E. coli* STa Enterotoxin Challenge

**Figure 1:**
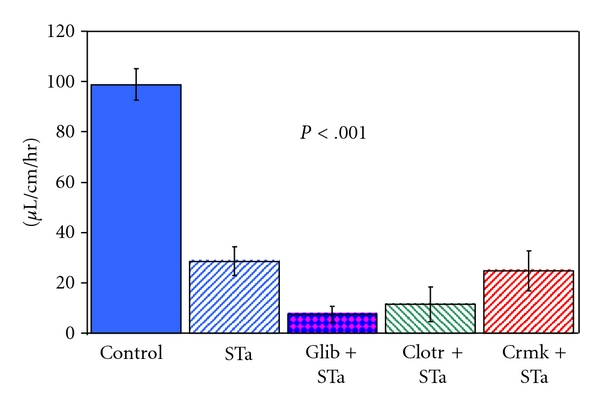
The effect on control jejunal fluid uptake (*n* = 14)   *in vivo* of luminal perfusion with 80 ng/mL *E. coli* STa alone (*n* = 17) and also STa in the presence of 300 uM (*n* = 6) luminal glibenclamide, 30 uM luminal clotrimazole (*n* = 4), and 1 uM luminal cromakalim (*n* = 10). Results are presented as the mean and standard error of the mean. Perfusion with STa caused a significant (*P* < .001) reduction in fluid uptake that was unaffected by the luminal addition of the potassium channel active agents.

When perfused without STa, the unchallenged jejunum (Figure 1) absorbed fluid at a rate of 98.8 ± 6.2 (14) *μ*L/cm/hr. In the STa challenged loop, there was a significantly lesser (*P* < .001) rate of 28.6 ± 5.8 (17) *μ*L/cm/hr absorption that was also significantly higher (*P* < .001) than zero net absorption. When glibenclamide was added to *E. coli* STa perfused loops at a concentration of 100 uM, known to normalise the increased membrane currents found in Caco2 cells after STa exposure [16], fluid absorption was 24.8 ± 8.9 (6) *μ*L/cm/hr which did not differ from absorption in the presence of STa without glibenclamide. This value of fluid absorption was still highly significantly (*P* < .001) below control rates of absorption, indicating that simultaneous glibenclamide perfusion had not improved the poor fluid absorption rates caused by STa enterotoxin. Glibenclamide at a concentration of 300 uM, known to reduce the short-circuit current increases in tracheal fibroblasts [17], also failed to restore fluid absorption, since this was 7.4 ± 3.2 (6) *μ*L/cm/hr, which was significantly (*P* < .05) below the rate in the presence of STa but without glibenclamide.

**Figure 2:**
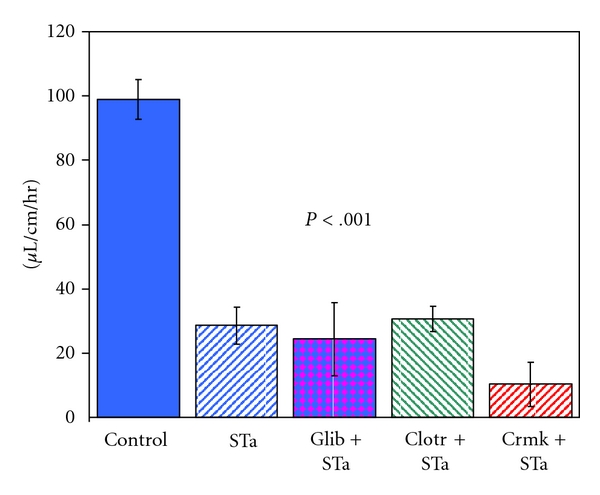
The effect of intravenous perfusion by jugular vein of glibenclamide (*n* = 6) at 7.0 mg/kg/hr, clotrimazole (*n* = 5) at 2.85 mg/kg/hr, and cromakalim (*n* = 5) at 2.72 mg/kg/hr on net fluid absorption from the proximal jejunum of the rat *in vivo* perfused luminally with 80 ng/mL *E. coli* STa heat stable enterotoxin. For comparison, fluid absorption without perfusion with STa (*n* = 14) and with perfusion with STa (*n* = 17) are given. Fluid absorption in the presence of STa was significantly lower (*P* < .001) than control values. I.v. perfusion of the indicated potassium channel active agents in the presence of STa was not significantly different from STa perfusion alone.

When glibenclamide was given intravenously (Figure 2), the mean fluid absorption rate with STa included in the perfusate was 24.4 ± 11.4 (6) *μ*L/cm/hr, not significantly different from the value of  28.6 ± 5.8 (17) *μ*L/cm/hr for luminal perfusion with STa without glibenclamide. Glibenclamide perfusion caused a noticeable pressor effect of approximately 16.7 ± 5.1 (5) mm Hg which indicated that while there was no effect on fluid uptake, the familiar effect of glibenclamide on mean arterial blood pressure was found.

### 3.3. Effect of Luminal and I.V. Clotrimazole on Luminal Fluid Absorption from the Proximal Jejunum after *E. coli* STa Enterotoxin Challenge

With a 30 uM concentration (known to inhibit completely the increment in short-circuit current after STa exposure *in vitro*) of clotrimazole in the lumen as well as STa, fluid absorption was 11.4 ± 7.0 (4) *μ*L/cm/hr, indicating that fluid absorption hindered by STa was not improved by inclusion of the potassium channel blocker. The i.v. perfusion of clotrimazole (Figure 2) during STa luminal perfusion was also without effect on fluid absorption. During clotrimazole perfusion, mean fluid absorption was 30.7 ± 4.0 (6) *μ*L/cm/hr during STa challenge, and this did not differ from the value of 28.6 ± 5.8 (17) *μ*L/cm/hr for fluid absorption in the presence of STa without simultaneous perfusion i.v. with clotrimazole. Clotrimazole was confirmed to have the anticipated depressor action on mean arterial blood pressure.

### 3.4. Effect of Luminal and I.v. Cromakalim on Luminal Fluid Absorption from the Proximal Jejunum after *E.coli* STa Enterotoxin Challenge

In the presence of 1 uM luminal cromokalim (Figure 1), STa reduced fluid absorption significantly (*P* < .01) to 24.7 ± 8.0 (10) *μ*L/cm/hr, which did not differ from the value for fluid absorption after STa challenge alone of 28.6 ± 5.8 (17) *μ*L/cm/hr. When cromakalim was administered intravenously (Figure 2), during STa perfusion, net fluid absorption significantly worsened to 10.3 ± 6.8 (5) *μ*L/cm/hr, indicating that fluid absorption after STa exposure was not restored to normal values but was worsened by cromakalim.

## 4. Discussion

A prevalent theory regarding secretion by the small intestine during diarrhoeal disease proposes that the enterocytes secrete chloride ion into the lumen. This is a necessary aspect of any hypothesis concerning an enterocyte origin for fluid secretion, because water movement occurs normally only in response to osmotic gradients. The mucosally secreted chloride and associated sodium ions purportedly increase osmolarity at the mucosal surface, causing fluid flow into the small intestinal lumen. To achieve chloride secretion from the enterocyte interior against the disadvantageous chemical gradient, this model incorporates enhanced potassium ion permeability to maintain a favourable electrical gradient. According to the enterocyte secretion concept, potassium ion exits across the basolateral membrane and maintains the mucosal membrane potential difference. As sustained secretion requires increased basolateral potassium ion permeability, fluid secretion should be inhibited by potassium channel blockers and possibly should also be accelerated by potassium channel openers.

Evidence for this particular aspect of the enterocyte secretion model derives largely from *in vitro* short-circuit current and ion efflux studies in tumour cells in Ussing chambers although neither parameter measures secretion as the mass transport of fluid. Prostaglandin *E *_1_ or* E. coli* STa increases a barium inhibitable rubidium flux across the serosal membrane, increases short circuit currents, and alters sodium and chloride fluxes across T84 cells [18, 19]. Potassium channel inhibitors such as glibenclamide are also CFTR inhibitors in tracheal fibroblasts [17] and should inhibit secretion. In addition, the potassium channel inhibitor clotrimazole inhibits elevated short-circuit current after forskolin treatment in T84 cells [5]. Clotrimazole does not affect mucosal membrane chloride conductance [4], and hence, its ability to prevent cholera toxin secretion is also attributed to altered potassium ion conductance. In addition, clotrimazole reduces the increased short-circuit current caused by cholera and *E. coli* STa enterotoxin [3]. Hence, *in vitro* evidence from electrical events and ion efflux studies points to potassium channel blockers preventing secretion.

In contrast, *in vivo* pharmacological tests of the role of enhanced enterocyte potassium ion permeability during enterotoxin challenge are rare, and the results are contradictory. Since clotrimazole [4] and glibenclamide [16] reduce the short-circuit current increase after STa challenge, these drugs should inhibit the action of this supposedly secretory enterotoxin. When tested *in vivo* by measuring loop* weight*, STa was modestly secretory, as was glibenclamide alone without toxin [6]. In contrast, cromakalim improved both normal absorption and also* E. coli* STa-mediated secretion despite being a potassium channel opener that should worsen or at least not improve secretion. Hence, neither agent affected fluid movement in the STa challenged jejunum in a manner predicted by the potassium ion permeability concept. The ability of cromakalim, the potassium channel opener, to prevent STa action and the ability of glibenclamide to cause secretion despite being a potassium channel blocker do not accord with the enterocyte potassium permeability model at all.

In the present experiments, neither luminal nor intravenous administration of clotrimazole, glibenclamide, or cromakalim affected the reduced fluid absorption occurring after exposure to *E. coli* STa enterotoxin. STa reduces fluid absorption severely, but if the potassium ion permeability model is valid, clotrimazole and glibenclamide should improve the poor fluid absorption, and cromakalim should perhaps worsen it. Although the theory predicts this, the observations of Shirgi-Degen and Beubler are at variance both with the present observations and with secretion theory. A three-hour perfusion time as used here allows large changes in volume to arise, since a reduction of 60 *μ*L/cm/hr in the rate of fluid absorption after STa challenge in a 25 cm loop translates into 4.5 millitres of fluid additionally present in the fluid reservoir at the end of experiment. Any net secretion and also any ability to inhibit secretion will be detected in this comparatively robust system and, in fact, was not found. Over a three-hour perfusion experiment, mean arterial blood pressure rose from 100 mm Hg to 120 mm Hg at the end of the experiment, indicating that mean arterial blood pressure is not compromised. Using the recovered volume technique, glibenclamide, cromakalim, and clotrimazole were shown not to have any ameliorating effect on STa induced reductions in fluid absorption.

To establish that the potassium channel modulators were active prior to intravenous administration or luminal perfusion, aliquots from the stock solutions were tested in separate, unrelated experiments. Intravenous injections of cromakalim and clotrimazole reduced mean arterial blood pressure *in vivo* in the rat, and glibenclamide had a moderate pressor effect, as others have found [11, 20]. These agents when given intravenously are unlikely not to have reached the enterocytes, since the diffusion path length from capillary to enterocyte is less than five micrometers. Intestinal capillaries, unlike normal capillaries, are known to have fenestrations that face the villi, and there are no substantial barriers to permeation. It is also unlikely that luminal perfusion did not achieve delivery to the enterocytes, since these drugs are lipid soluble with dissociation constants that allow absorption. It might be argued that secretion of fluid may keep luminally administered drug off the surface, but given the diffusion coefficients of molecules of this size, the required adverse convection currents can be calculated to be orders of magnitude above hypothesised rates of secretion. In addition, the concentrations of dimethyl sulphoxide and ethanol are known to have no effect on STa-mediated guanylate cyclase activation [21]. Confirming this, luminal STa reduced fluid absorption in all experiments regardless of presence of vehicle. Where vehicle alone was perfused as a control experiment, no effect on STa action was noted. The lack of effect of potassium ion channel active compounds is not because of interference from dispersant vehicle and highly unlikely to be because of inability to reach the enterocyte.

The cardiovascular actions of the selected agents may explain differences between this study and that of Shirgi-Degen and Beubler [6]. Luminal volume as measured by the fluid recovery can only reflect secretion or absorption across the luminal membrane. In contrast, any method based on loop *weight* also measures additionally fluid entering and leaving the vasculature, causing changes in the interstitial volume. Since measurement of loop weight also includes interstitial volume, which the luminal fluid recovery method does not, differing findings might result from vasomotor changes. This might explain why potassium channel active agents do not alter fluid absorption after STa challenge when assessed by luminal volume recovery in the present experiments but seem to reduce secretion when this is defined and measured as an increase in loop weight. This could also explain why cromakalim is antagonized by glibenclamide in the Beubler study, as a depressor and a pressor substance compete to alter peripheral resistance and hence intestinal interstitial volume. If these changes are not explicable by local vascular changes within the intestine, then any explanation defaults to the level of the enterocyte. 

Since these compounds do not inhibit fluid movement in the STa challenged intestine when the recovered volume technique is used, then the potassium channel activation model does not apply despite the availability of *in vitro* evidence indicating that it should be relevant. One explanation for the *in vitro* results is that rubidium efflux and short-circuit-current changes relate to the sodium: potassium pump rate and not to chloride secretion. The known inhibition by clotrimazole of the sodium pump may explain the fall in short circuit current in isolated cells [22]. Similarly, enhanced potassium loss from the cells may arise from the sodium pump working at a faster rate to re-establish normal rates of fluid absorption when the cell is challenged by bacterial enterotoxin. This could explain the undoubted effects of potassium channel active agents on electrical current without any real effect on fluid movement.

In conclusion, potassium ion active agents do not restore *in vivo* the reduced net fluid movement caused by *E. coli* STa enterotoxin, widely regarded as causing secretion. Enhanced potassium ion permeability to maintain the electrical gradient for adequate luminal exit of chloride ion is a sub-hypothesis of the chloride secretory model [23] rather than a necessary corollary. Falsification of this concept alone does not itself falsify the chloride secretion hypothesis for fluid movement into the intestine. However, the action of *E. coli* STa enterotoxin is not prevented by potassium channel modulators despite promising data from *in vitro* techniques that are assumed to but may not measure secretion. The lack of effect of potassium channel modulators is further evidence that STa is not a secretory toxin but acts solely by inhibiting sodium ion and fluid absorption.

## Figures and Tables

**Table 1:** The pressor and depressor effects of clotrimazole (1 mg/kg i.v.), glibenclamide (1.5 mg/kg i.v.), and cromakalim (8–24 ug/kg i.v.) on the systolic, diastolic, pulse, and mean arterial blood pressure in the anaesthetised rat. Blood pressures are expressed as mm Hg tension. Results are expressed as means ± standard error of the mean with the number of observations in parenthesis or as differences in the means before and after intravenous drug administration. Statistical significance (^a^ *P* < .001; ^b^ *P* < .02; ^c^ *P* < .05) for comparison of parameters prior to and after drug administration.

	Systolic pressure (mm Hg)	Diastolic pressure (mm Hg)	Pulse pressure (mm Hg)	Mean arterial pressure (mm Hg)
Control	128 ± 12 (6)	112 ± 14 (6)	26 ± 3 (6)	117 ± 14 (6)
1 mg/kg clotrimazole	87 ± 8 (6)^b^	66 ± 6 (6)^a^	27 ± 6 (4)	73 ± 13 (6)^a^

Control	116 ± 12 (6)	93 ± 14 (6)	23 ± 2 (6)	101 ± 13 (6)
8 ug/kg cromakalim	116 ± 9 (3)	94 ± 11 (3)	22 ± 2 (3)	101 ± 10 (3)
16 ug/kg cromakalim	98 ± 8 (3)	71 ± 9 (3)^c^	27 ± 3 (3)	80 ± 9 (3)^c^
24 ug/kg cromakalim	76 ± 9 (3)^a^	49 ± 3 (3)^a^	27 ± 6 (3)	58 ± 5 (3)^a^

Control	103 ± 6 (7)	77 ± 7 (7)	26 ± 4 (7)	86 ± 6 (7)
1.5 mg/kg glibenclamide	126 ± 10 (7)^c^	99 ± 10 (7)	26 ± 4 (7)	109 ± 10 (7)

**Table 2:** The effect of 100 uM clotrimazole, 50 uM glibenclamide, and 7 uM cromakalim on the tension and rate of smooth muscle contraction expressed as grams tension in the *in vitro* rabbit ileum incubated in Krebs-Ringer solution. Results are expressed as means ± standard error of the mean with the number of observations in parenthesis. Statistical significance (^a^ *P* < .001; ^b^ *P* < .02; ^c^ *P* < .05) for comparison of parameters prior to and after drug administration.

	Basal tension (gms)	Peak tension (gms)	Average tension (gms)	Tension amplitude (gms)	Rate of contraction (per minute)
Pre clotrimazole	2.51 ± 0.88 (4)	5.90 ± 0.76 (4)	3.74 ± 0.53 (6)	3.39 ± 0.88 (4)	13.9 ± 1.1 (4)
Post clotrimazole	2.50 ± 0.84 (4)	4.63 ± 0.55 (4)^a^	2.61 ± 0.72 (6)^c^	2.18 ± 0.81 (4)^a^	13.4 ± 1.5 (4)

Pre glibenclamide	1.98 ± 0.54 (3)	6.69 ± 0.79 (3)	4.34 ± 0.41 (3)	4.70 ± 1.07 (3)	14.6 ± 2.4 (3)
Post glibenclamide	3.14 ± 0.57 (3)^a^	8.01 ± 2.45 (3)	5.58 ± 1.51 (3)	4.87 ± 1.90 (3)	13.8 ± 2.6 (3)

Pre cromakalim	1.73 ± 0.62 (3)	6.50 ± 0.81 (3)	4.12 ± 0.26 (3)	5.10 ± 1.33 (3)	14.5 ± 1.8 (3)
Post cromakalim	1.60 ± 0.64 (3)	4.37 ± 1.62 (3)	2.98 ± 1.06 (3)	2.77 ± 1.25 (3)^c^	13.8 ± 2.1 (3)
